# Supplementary material for: Role of a FAD-dependent monooxygenase in diazo group functionalization of kinamycin in Streptomyces ambofaciens
Source: Microbiology (Reading). 2025 Jun 20;171(6):001576. doi: 10.1099/mic.0.001576 (PMC12181624; doi:10.1099/mic.0.001576)
Supplement: Uncited Supplementary Material 1. [file mic-171-01576-s001.pdf]

Supplementary information

Role of a FAD-dependent monooxygenase in diazo group functionalisation of kinamycin in *Streptomyces ambofaciens*

Cláudia M. Vicente[1*], Alexis Boutilliat[1], Laurence Hôtel[1], Cédric Paris[2], Bertrand Aigle[1*]

[1]Université de Lorraine, INRAE, DynAMic, 54000 Nancy, France

[2]Université de Lorraine, LIBio, 54000, Nancy, France

[*]Correspondence:
claudia.vicente@inrae.fr
bertrand.aigle@inrae.fr

Table S1. Strains and plasmids used in this study.

Strains / plasmids	Relevant properties[a]	Source
S. ambofaciens ATCC23877	wild-type strain	[1]
S. ambofaciens ΔΔ*alp2F*	SAM23877_0158 locus replaced by the *aac(3)IV-oriT* cassette	This study
S. ambofaciens ΔΔ*alp2F* /pRT802:: *alp2F-alp2G*	mutant strain complemented with *alp2F*	This study
E. coli		
DH5α	cloning strain	[2]
BW25113	strain used for gene replacement/mutation	[3]
ET12567/pUZ8002	donor strain in interspecific conjugation	[4, 5]
B. subtilis ATCC6633	indicator strain for bioassay antibiotic detection	
Plasmids/cosmids		
E8	*S. ambofaciens* ATCC23877 genomic library cosmid	[6]
pIJ773	*oriT*, *aac(3)IV*	[7]
pIJ790	*oriT*, *exo*, *cat*, *gam*, *bet*	[7]
E8Δ*alp2F*::*aac(3)IV*	*alp2F* is replaced by *aac(3)IV-oriT*	This study
pRT802	ϕBT1-integrative plasmid, *neo*, *attP*	[8]
pRT802::*alp2F-alp2G*	pRT802-derived plasmid with the SAM23877_0158-SAM23877_0157 locus	This study

[a] *aac(3)IV*, apramycin resistance gene; *cat*, chloramphenicol resistance gene; *neo*, kanamycin resistance gene; *gam*, inhibits host exonuclease V; *bet*, single-stranded DNA binding protein; *attP*, attachment site of φBT1; *oriT*, origin of transfer.

Table S2. Primers used in this study.

Name	Sequence (5’-3’)	Function
**amont_creE**	gccgccgagcacaggaacatggcgggcaccgggcacatgATTCCGGGGATCCGTCGACC	selection marker amplification for mutant construction
**aval_creE**	ggcaggcggggccggcgggggcgggctggtcgcggctcaTGTAGGCTGGAGCTGCTTC	
**creED_amont**	CATAggatccCCCTCCTCCTCCTGGACG	mutation verification and locus amplification for plasmid construction
**creED_aval**	ACAGgaattcGGTCTGAGCAACTTGTGGC	

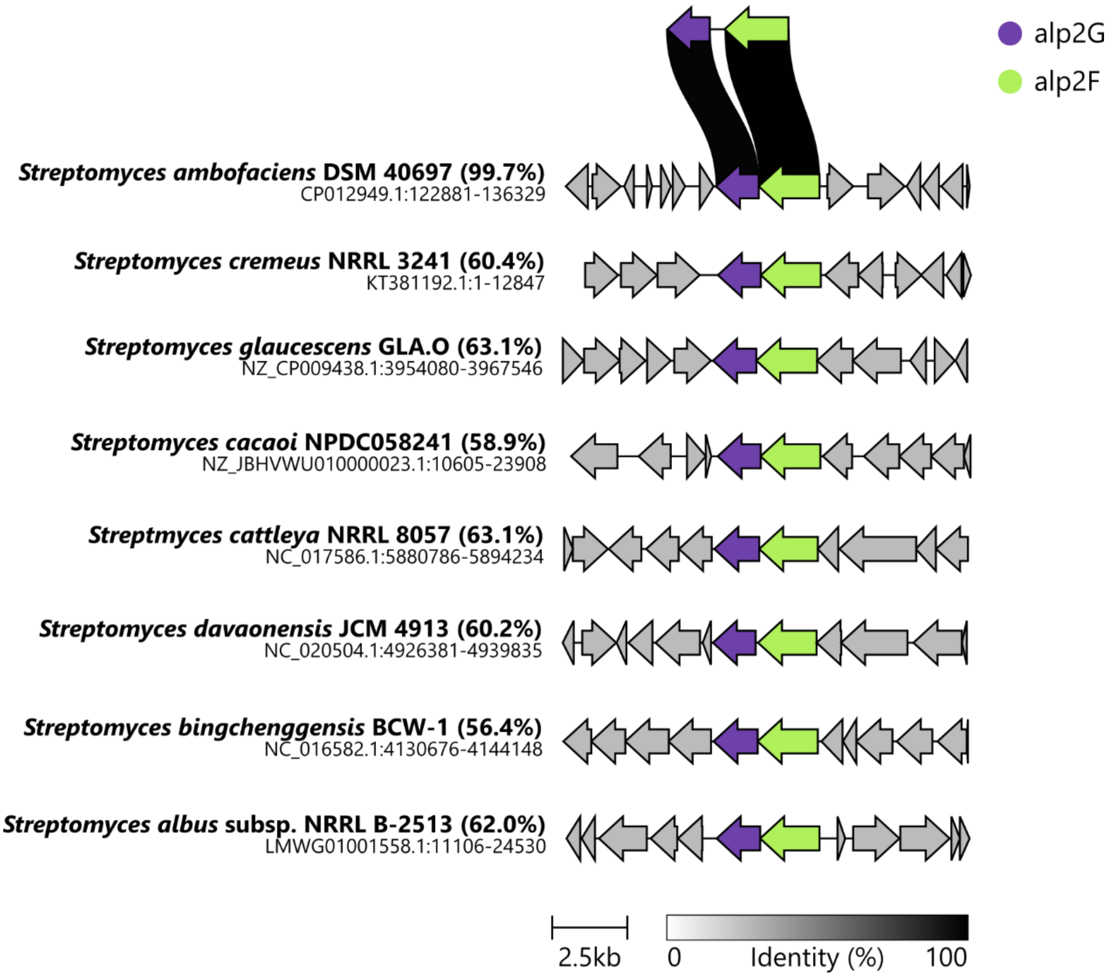

Figure S1. Alignment of clusters containing *alp2F* and *alp2G*-like genes found in *Streptomyces*. Identity percentage comparing *alp2F* from *S. ambofaciens* ATCC23877 with each of the other shown strains.

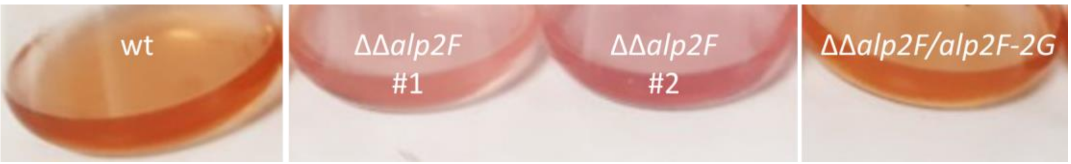

Figure S2. The presence of *alp2F* gene results in differently coloured fermentation extracts. The crude extracts obtained using an acidified extraction solution from the mutant strains ΔΔ*alp2F* (99% ethyl acetate and 1% acetic acid; see Materials and Methods) present a pink colour compared to the orange wild-type extracts. The orange coloration phenotype is restored when the gene is reintroduced in the complemented strain ΔΔ*alp2F*/*alp2F*-*alp2G*.

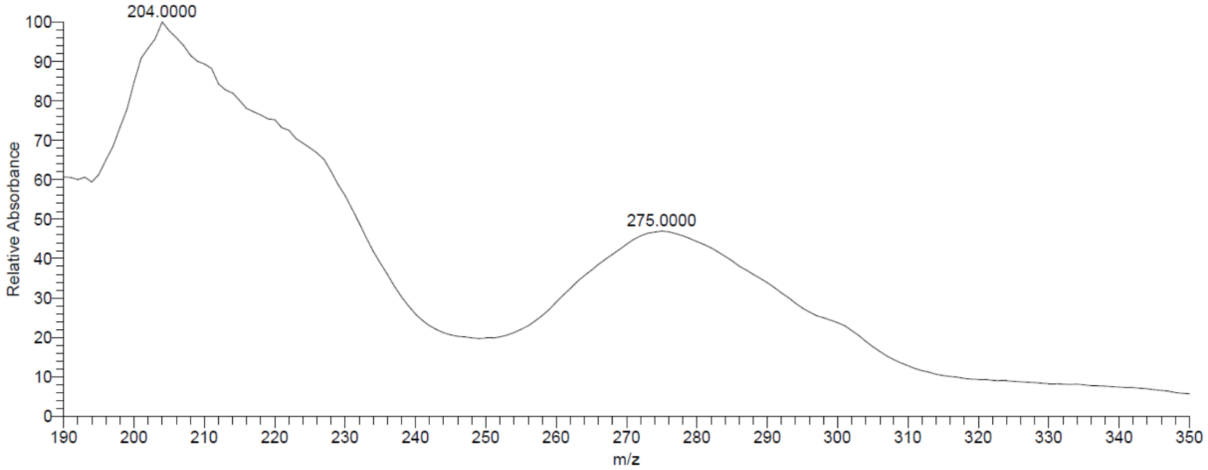

Figure S3. UV absorption spectrum of peak identified as stealthin C.

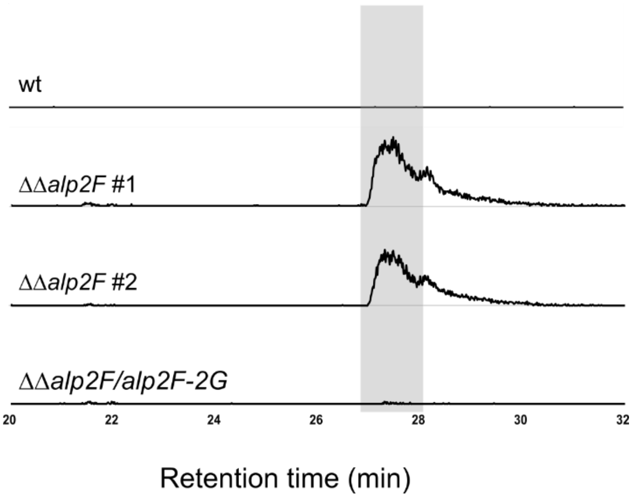

Figure S4. LC-MS analysis of other metabolites in the kinamycin biosynthetic pathway in the different strains. Seongomycin at m/z=454.0960 under its monoprotonated form (highlighted in gray) was detected in the mutant strain ΔΔ*alp2F* but not in the wild-type or complemented strain ΔΔ*alp2F/alp2F-2G*.

References

1. **Pinnert-Sindico S**. Une nouvelle espèce de Streptomyces productrice d'antibiotiques: Streptomyces ambofaciens n. sp., caracteres culturaux. *Ann Inst Pasteur* 1954;87:702–7.

2. **Hanahan D**. Studies on transformation of Escherichia coli with plasmids. *J Mol Biol* 1983;166:557–580.

3. **Datsenko KA, Wanner BL**. One-step inactivation of chromosomal genes in Escherichia coli K-12 using PCR products. *Proc Natl Acad Sci U S A* 2000;97:6640–6645.

4. **MacNeil DJ**. Characterization of a unique methyl-specific restriction system in Streptomyces avermitilis. *J Bacteriol* 1988;170:5607–5612.

5. **Paget MS, Chamberlin L, Atrih A, Foster SJ, Buttner MJ**. Evidence that the extracytoplasmic function sigma factor sigmaE is required for normal cell wall structure in Streptomyces coelicolor A3(2). *J Bacteriol* 1999;181:204–211.

6. **Leblond P, Fischer G, Francou FX, Berger F, Guérineau M, *et al.*** The unstable region of Streptomyces ambofaciens includes 210 kb terminal inverted repeats flanking the extremities of the linear chromosomal DNA. *Mol Microbiol* 1996;19:261–271.

7. **Gust B, Challis GL, Fowler K, Kieser T, Chater KF**. PCR-targeted Streptomyces gene replacement identifies a protein domain needed for biosynthesis of the sesquiterpene soil odor geosmin. *Proc Natl Acad Sci U S A* 2003;100:1541–1546.

8. **Gregory MA, Till R, Smith MCM**. Integration site for Streptomyces phage phiBT1 and development of site-specific integrating vectors. *J Bacteriol* 2003;185:5320–5323.
